# A mixed methods evaluation of peer support in Bristol, UK: mothers’, midwives’ and peer supporters’ views and the effects on breastfeeding

**DOI:** 10.1186/1471-2393-13-192

**Published:** 2013-10-20

**Authors:** Jenny Ingram

**Affiliations:** 1 Centre for Child and Adolescent Health, School of Social and Community Medicine, Oakfield House, Clifton, Bristol BS8 2BN, UK

**Keywords:** Breastfeeding, Peer support, Self-efficacy, Survey, Qualitative, Evaluation

## Abstract

**Background:**

International studies suggest that breastfeeding interventions in primary care are more effective than usual care in increasing short and long term breastfeeding rates. Interventions that combine pre- and postnatal components have larger effects than either alone, and those that including lay support in a multicomponent intervention may be more beneficial. Despite the mixed reports of the effectiveness of breastfeeding peer support in the UK, targeted peer support services are being established in many areas of the UK. In 2010, NHS Bristol Primary Care Trust commissioned a targeted breastfeeding peer support service for mothers in 12 lower socio-economic areas of the city, with one antenatal visit and postnatal contact for up to 2 weeks.

**Methods:**

Mothers receiving the peer support service were invited to complete an on-line survey covering infant feeding; breastfeeding support; and confidence in breastfeeding (using the Breastfeeding Self-Efficacy Scale). Semi-structured interviews and a focus group explored perceptions of mothers, midwives and peer supporters. The effects of the service on breastfeeding rates were documented and compared.

**Results:**

163 mothers completed the on-line survey; 25 participants were interviewed (14 mothers, 7 peer supporters and 4 maternity health professionals); exclusive and total breastfeeding rates for initiation and at 8 weeks were compared for 12 months before and after the service started.

The targeted peer support service was associated with small non-significant increases in breastfeeding rates, (particularly exclusive breastfeeding), compared to the rest of the city. The service was very positively evaluated by mothers, health professionals and peer supporters. Mothers felt that peer support increased their confidence to breastfeed; peer supporters found the contacts rewarding, enjoyable and important for mothers; midwives and maternity support workers were positive about the continuity of an antenatal visit and postnatal support from the same local supporter.

**Conclusions:**

The introduction of a targeted peer support service was associated with psycho-social benefits for mothers, health professionals and peer supporters. Continuity of peer support with an antenatal visit and postnatal support from the same local supporter was also thought to be beneficial.

## Background

A systematic review of international studies of breastfeeding interventions in primary care reported that they are more effective than usual care (without such interventions) in increasing short and long term breastfeeding rates [1]. Interventions that combined pre- and postnatal components had a larger effect than either alone, and those that included lay support (such as peer support) in a multicomponent intervention may be more beneficial. A Cochrane systematic review of the effectiveness of breastfeeding support concluded that all forms of extra support (from trained peers or professionals) showed an increase in the length of time women continued to breastfeed; support by both lay supporters and professionals had a positive impact on breastfeeding outcomes; and face-to-face support was associated with a larger effect than telephone support [2]. However similar reviews of breastfeeding peer support interventions during pregnancy and the postnatal period have indicated that they only have small effects on breastfeeding rates in the UK [3-5]. In low or middle income countries, peer support has been shown to increase breastfeeding continuation rates, especially exclusive breastfeeding, but in countries where there is routine postnatal healthcare it does not have such a marked effect [3,6]. Hoddinott [7] in an evidence synthesis of nine UK randomised trials of breastfeeding interventions explored these issues and has highlighted the complex nature of the interventions, the importance of the context in which an intervention is delivered, the choice of intervention, and the nature of the behaviour change involved. All these factors can contribute to an apparent absence of the effect of an intervention.

Universal antenatal peer support does not appear to increase breastfeeding initiation rates, but targeted peer support may be beneficial [4]. The role of the peer supporter seems to be most important in the postnatal period and peer supporters can provide complementary support to that provided by health professionals [8].

Studies have explored women’s reflections on breastfeeding peer support using concept analysis and particularly the concept of hope and how peer supporters help women focus their energy to achieve their breastfeeding goals [9]. Wade [10] suggested that breastfeeding peer support could improve women’s mental health, parenting skills, and increase self-esteem and confidence. Kempenaar & Darwent [11] showed that accredited peer support training improved breastfeeding knowledge and attitudes of mothers who undertook it and enabled them to facilitate informed choices about breastfeeding and provide effective support for breastfeeding. Other peer support initiatives in areas of economic deprivation have used a combination of peer support with breastfeeding support groups to increase breastfeeding prevalence [12,13]. A comparison of one-to-one or group-based peer support in rural Scotland however, concluded that group-based peer coaching was more successful in that context as women perceived that one-to-one coaching was a greater risk to their confidence and empowerment than attending a group [14].

Despite these mixed reports, targeted peer support services are being established in many areas of the UK to try to improve the low breastfeeding rates at 6 months, particularly exclusive breastfeeding, as less than 1% of babies are exclusively breastfed at 6 months in the UK [15]. One such peer support service based in areas of low breastfeeding prevalence in a city in South-West England has been established. This evaluation documented the effects of the service on breastfeeding rates and explored the perceptions of mothers, midwives and peer supporters.

### Context

NHS Bristol Primary Care Trust commissioned a leading children’s charity (Barnardo’s: http://www.barnardos.org.uk) to provide a targeted breastfeeding peer support service for mothers (in 12 areas of low breastfeeding prevalence in the city) with one antenatal visit and postnatal contact at 48 hours after coming home which continued for 2 weeks. The service (Bristol Breastfeeding Peer Support Service) aimed to meet UNICEF/WHO Baby Friendly Initiative (BFI) guidance on the provision of antenatal information, and NICE guidance [16] on the provision of peer support contact in the 48 hours after discharge from hospital. It also aimed to provide intensive support to breastfeeding mothers at the time when there are high levels of attrition. In the areas of the city involved in the service there were also two voluntary breastfeeding counsellors (one from La Leche League, the other from the Association of Breastfeeding Mothers) who were available for mothers to contact, but they were mainly accessed at local breastfeeding support groups.

The peer supporters’ training was accredited by La Leche League and comprised 10 sessions of 2.5 hours each initially, with extra Safeguarding and Lone Working sessions added later. It covered anatomy and physiology, babies’ needs, positioning and attachment, motivational interviewing and general communication skills for antenatal meetings and postnatal follow-up, making breastfeeding work and record keeping. These skills were used extensively during the antenatal visit, which often included the woman’s partner or her own mother, and during contacts made postnatally.

Names of all pregnant women in the relevant areas were collected by the peer supporters from local community midwives on a weekly basis. Sharing data between the midwives and the charity in this way was agreed by all parties as the best method for collecting the names. In some areas of Bristol, trained maternity support workers (MSWs) were employed to support community midwives by providing postnatal women with breastfeeding support and they were often involved in this process. The role of MSWs in the UK has been described elsewhere [17].

A Barnardo’s paid peer supporter, based in each of the 12 areas, contacted all the women in her area when they were around 32 weeks’ gestation to offer a home visit to talk about infant feeding choices and the support available for breastfeeding. The same supporter also contacted the mother by phone 48 hours after she went home after the birth to offer further support and information about local breastfeeding support groups. Women could opt out of the service or the evaluation at any time. The number of peer supporters decreased from twelve to eight over the first few months of the service and remained at this level for most of the evaluation period.

## Methods

At the first antenatal contact, mothers were told about the evaluation and asked if they had an email address, as the evaluation survey would mainly be conducted on-line. At the 48 hour postnatal contact they were reminded about the questionnaire survey. Two weeks later Barnardo’s sent each mother an email inviting her to complete the on-line survey. The email message included an opt-out of both the survey and further evaluation at this point. A few participants preferred to be contacted by telephone. Email addresses of those agreeing to the survey were sent to the researcher, who sent reminders to those who had not completed it two weeks later and telephoned those without email. The on-line survey was hosted by Bristol Online Survey (BOS) and the data stored on a secure server at the University of Bristol.

The survey questions were based on questions used in previous evaluations of breastfeeding support [13,18,19] and agreed by JI with two local breastfeeding specialists. The questions included demographic information about the mother and baby, feeding history, use of skin to skin contact, breastfeeding support, the antenatal visit, postnatal contacts, sources of further support and free text comments. Mothers also completed a self-efficacy questionnaire (the Breastfeeding Self-Efficacy Scale- short form: BSES-SF) which comprises 14 five-point Likert-scale questions with a total score of 70. Theoretically based on Bandura’s social cognitive theory [20], the BSES-SF is an instrument that measures a mother’s confidence in her ability to breastfeed her new baby [21]. It can be used clinically to identify breastfeeding mothers at high risk of discontinuing breastfeeding and to assess breastfeeding behaviour. Research has also shown that women who feel self-confident about their ability to breastfeed successfully are more able to overcome social barriers to breastfeeding [22]. BSES-SF scores were compared statistically by feeding status and parity using Chi-square tests.

At the end of the survey, mothers were asked if they would be prepared to have a short telephone interview to explore their views of the service and peer support in more detail. Questions explored included their feelings on the antenatal visit, who else was involved and how involved they were; postnatal topics included the nature, frequency and helpfulness of the peer supporter contacts and any other services used as well as their overall views of the peer support service. A purposive sample of women was selected, from those who provided their contact details, to include a wide range of postcodes and dates of birth of babies from across the evaluation period.

Midwifery teams (midwives and MSWs) in two areas were also contacted and invited for interview (by telephone) to explore their experiences of the service, how it was working both antenatally and postnatally, if there had been any problems or barriers to overcome, how the peer supporters fitted into the midwifery team and thoughts for the future of the service.

A focus group of the paid peer supporters was held in December 2011, just over one year after the start of the service, and was facilitated by JI. Topics discussed included their views and challenges overcome in doing antenatal visits, the postnatal contacts with women, relationships with NHS staff, and their feelings about the service as a whole.

All the interviews and the focus group were digitally recorded, transcribed, anonymised and checked for accuracy before analysis. Thematic analysis using an inductive approach [23] was used to scrutinise the data to identify and analyse patterns across the dataset and this process was led by the main researcher. Following repeated reading of the transcripts, codes were generated and built into themes and sub-themes [24]. Final themes were discussed and refined within the evaluation team to achieve a coding consensus and ensure robust analysis.

A concurrent triangulation mixed methods approach [25] was used to combine and compare the views of mothers, peer supporters and health professionals and to integrate the free texts comments from the survey with the semi-structured interviews. This type of mixed methods analysis was used to add to the richness of the data and integrate insights from the different parts of the study to produce a single narrative.

Breastfeeding rates (both exclusive and any breastfeeding) for all areas of Bristol were collated prospectively by the public health analysts at NHS Bristol, from data supplied by the Bristol NHS maternity provider trusts and 8-week check data collected by the Avon Child Health Surveillance system (routine data collected by GPs). Barnardo’s collated the referral rates of women to the Bristol Breastfeeding Peer Support Service and breastfeeding information at the two week cut off point for the service. Statistical comparisons between rates for the 12 target areas and the rest of Bristol were made using chi-square tests.

Ethical approval was granted by the University of Bristol Faculty of Medicine and Dentistry Committee for Ethics in August 2010. The women involved in the study opted into the study by responding to the survey and subsequently volunteered to be interviewed by providing their contact details at the end of the survey. Ethical principles to ensure informed consent, autonomy and confidentiality were adhered to in the evaluation.

## Results

### Survey participants

The survey ran for 12 months from the end of 2010 to the end of 2011 and a total of 426 names of women who had received peer support were sent by email to the evaluation team. Of the 426, 99.3% (423) agreed to take part in the evaluation and 78% (330) of them had email addresses; 93 preferred contact by telephone. After the initial email from Barnardo’s, 71% (234) received a reminder email two weeks later, and a further attempt was made to reach those without email by telephone. 163 (38.5%) surveys were completed, most of which were self-completed by those with email addresses (153: 46.4%).

Table 1 shows details of the mother’s age, baby age when the survey was completed, age when breastfeeding stopped (if relevant) and mothers’ confidence with breastfeeding recorded from the Breastfeeding Self-Efficacy score.

**Table 1:** Demographics of survey mothers (n = 163)

	**Mean**	**Median**	**Range**
Baby age when survey completed	30 days	34 days	14 – 42 days
Mother’s age	29.6 years	30 years	16 – 40 years
Breastfeeding self-efficacy score (maximum score =70)	52	55	14 - 70
Baby age when stopped breastfeeding (n = 27 mothers)	15.8 days	14 days	1 – 30 days

Most of the mothers (93%) lived with a partner and 11 were single parents; for 66% of the mothers this was their first baby and 53% of the babies were boys. All those completing the survey started breastfeeding; 62% (101) were still exclusively breastfeeding when they completed the survey and a further 21% (35) were mixed feeding. 42% of mothers reported that their partners had been most helpful in supporting breastfeeding, 29% felt that the peer supporter had helped them most, 15% mentioned their midwife and some said that it was a combination of all three with the local breastfeeding counsellors also involved. Almost all women had had close ‘skin to skin’ contact with their baby for periods of time; for 24% this was only for a period after birth, but 74% were still having this close skin contact several weeks after the birth. This was something that peer supporters encouraged mothers to do to promote and support breastfeeding.

### Peer supporter contacts

Most women (96%) reported that they had received an antenatal visit from their peer supporter. Table 2 shows that many found the visit helpful, encouraging, welcome and clear, and the postnatal calls received were also helpful, supportive and reassuring. The timing of the first postnatal contact was felt to be ‘at the right time’ for 91% of women; but a few (7%) felt that it had been a bit too late for them.

**Table 2:** How women in the survey described the antenatal visit and postnatal phone calls

**Antenatal visit description (n = 163)**	Number (%*)	**Postnatal phone calls (n = 163)**	Number (%*)
Helpful	129 (79%)	Helpful	103 (63%)
Encouraging	82 (50%)	Supportive	80 (49%)
Welcome	60 (37%)	Welcome	66 (40.5%)
Clear	43 (26%)	Reassuring	60 (37%)
Unhelpful	1 (0.6%)	Unhelpful	2 (1%)
Confusing	1 (0.6%)	Inconvenient	2 (1%)
Annoying	0	Worrying	1 (0.6%)
Discouraging	0	Annoying	0

*percentages do not add up to 100% as more than one adjective could be selected.

At the antenatal visit, 68 partners had attended the meeting and 10 mothers (of the pregnant women). Women reported that their partners/mother felt included in the meeting, were able to ask questions and found the answers helpful. For those who mentioned that they found the peer supporter their most helpful breastfeeding supporter, 52% of their partners had attending the antenatal meeting, perhaps suggesting that their partner may have encouraged contact with the peer supporter, whom they had met previously.

Following the initial postnatal calls from the peer supporters, 31% of women had continued to keep in touch with their peer supporter for advice; 36% mentioned that they had been to a local breastfeeding support group for breastfeeding help after being signposted by the peer supporter; and 23% had contacted a volunteer breastfeeding counsellor.

### Breastfeeding self-efficacy

Responses to the Breastfeeding Self-efficacy questionnaire showed that mothers who were exclusively breastfeeding when they completed the survey had the highest self-efficacy scores (mean score 58.4, SD 11.6), followed by those who were mixed feeding (mean score 48.5, SD 11.4). Those who were not still breastfeeding completed it to reflect how they felt when they had last breastfed (mean score 30, SD 12.5). Those who were feeding their second or subsequent baby were more confident than those breastfeeding for the first time, and those who were exclusively breastfeeding at the time of completing the survey were also significantly more confident (Chi-square test p < 0.001). These scores are very similar to those found by others who have shown that early confidence in breastfeeding is associated with longer duration of breastfeeding [26].

### Interviews

48 women gave their contact details in the survey as volunteering for an interview and 14 semi-structured interviews were conducted with parents; 13 by telephone and one face to face. These interviews explored the advice, help and support that mothers and their partners had received in more detail at the antenatal visit and in the postnatal contacts. At the time of the interview the babies were between 2 and 4 months old, nine were breastfed, one mixed fed and four were formula fed. Telephone interviews were conducted with 6 midwives and two MSWs; and seven of the eight paid peer supporters attended the focus group.

### Views of parents, peer supporters and health professionals

Four main themes arose from the thematic analysis within the context of the topics explored in the interview schedules: antenatal opportunity for knowledge; postnatal reassurance; encouragement and self-confidence; and the challenges of peer support.

### Antenatal opportunity for knowledge – ‘it was informal with time for discussion’

All mothers enjoyed the antenatal visit which gave them opportunities to learn more about breastfeeding and some had their partner or mother at the visit. They praised the depth of information given, but that it was also informal with plenty of time for discussion.

“It was quite comfortable because it was informal, I liked the tone and it was helpful as well”. (Mother #1, first baby)

“Personally I found it extremely helpful, you think it is a natural thing but it’s not and I was very anxious about it and (PS) came to see me and she was here about an hour and she answered all my questions and after that visit I felt so much better and more confident”. (Mother #11, second baby, first time breastfeeding)

“Very helpful to answer questions the midwives did not have the time to go into; this makes a real difference in terms of motivation to continue breastfeeding”. (survey mother #199)

Fathers felt involved in the discussion and could ask questions:

“Yes well I just kept quiet for a bit, and then she told us about the size of the baby’s stomach over a period of time, that was interesting,… she brought a knitted breast and doll to show how to breastfeed......I think it was the first chat that we’d had with a third party, I suppose, and so for the first 10 minutes I just let her chat, but she was just really easy to talk to and very friendly and nice”. (Father #4 of twins)

Peer supporters felt that the antenatal meeting was really worthwhile and important for mothers and described the visits as being ‘great’, ‘brilliant’, ‘enjoyable’! Women appeared to be keen to breastfeed when they talked to them at the antenatal visit and by attending and talking to women at antenatal clinics, peer supporters were able to include visits to more women and not just those who were planning to breastfeed. They reported that some women managed to do a few breastfeeds in hospital, which they might not have bothered with before.

“Yes I think some have done the first few feeds in hospital which they might not have done without our chat and if they had some support in hospital they have given it a go. One woman having her 4th baby said that I had really opened her eyes and that she was going to ‘give it a go’ with this baby as she has a more realistic picture of what breastfeeding is about”. (PS #2)

Midwives commented that they felt that the peer supporter antenatal contacts were a good thing and the MSWs felt that peer support complemented and enhanced their own role and they were confident about what the peer supporters were doing. One had accompanied a peer supporter on her antenatal visit:

“I was very impressed with how the discussion was conducted, it was absolutely brilliant!” (MSW #1)

### Postnatal reassurance – ‘someone there for me’

Initial peer supporter contact postnatally was often by text followed by phone calls and sometimes visits if mothers were struggling with breastfeeding. Mothers commented on the reassurance and helpful advice provided by the peer supporters, which helped them to overcome difficulties and continue to breastfeed, and the convenience of keeping in touch by text.

“We did most of our communication by text, it worked really well. You can write texts whenever you have time, stop and start. Sometimes she used to text me to say that she had sent an email with some information in that might be useful. Then she also came round to see us”. (Mother #13, first baby)

“It was really nice .. because I knew that my sister had struggled with breastfeeding, there was a real sense of peace knowing that there was someone there and someone who I could contact and that was interested”. (Mother #4 of twins)

“Any difficulties at home I had (PS) to turn to, she was fantastic, she was reassuring, made good suggestions. I felt I could ring her and say I was struggling, it was quite hard at times, she gave me that level of support that I could carry on. Without that I would have struggled to carry on, I really can’t rate it highly enough. My husband was also very grateful that there was somebody to turn to and that was reassuring for him. (Mother #6, second baby)

“Brilliant! The main thing I appreciated was being able to text someone I knew with a question or concern and know that they would call back with advice and support. This is definitely preferable to calling a general phone helpline (eg La Leche League or National Childbirth Trust), which is far more unknown”. (survey mother #106)

“Brilliant fantastic service - this is my 7th baby but the first one that I have breastfed. I would definitely recommend it to everyone and breastfeeding too!” (survey mother #137)

“The PS was very helpful, gave me lots of time to talk and ask questions. It's a good service and should be given to all. I wish my friends had had that input as some were made to feel that they weren't doing it right and they gave up”. (survey mother #147)

Midwives mentioned complementary support from the peer supporters as important and MSWs felt that they complemented and enhanced their own role and were confident about what the peer supporters were doing. They appreciated the service and felt that the ‘joined up support’ had kept some women breastfeeding for longer.

“We feel that peer supporters have a clear and complementary role to play alongside the midwifery team”. (MW #1)

Some mothers also mentioned the complementary support provided by their peer supporter with other support available which works well in some areas.

“I think with the supporter, breastfeeding counsellor and health visitor, yes, they all worked well, … it was the supporter and the breastfeeding counsellor, they kept me going really, getting the technique right I could have quite easily gone onto the bottle quite quickly with all the troubles that I had”. (Mother #10, first baby)

### Encouragement and enhanced self-confidence

Women commented about the importance of providing this support for all mothers and how it had given them confidence to continue to breastfeed, which their friends had not received and had often given up breastfeeding.

“I feel very strongly that this useful and practical advice given in the comfort of your own home environment in those very early days was an invaluable support. I can only believe that if more women were given this support there would be much more tendency to breastfeed. I just wanted to say how much it has made a difference to me and how much I valued the breastfeeding support provided by the peer supporter”. (Mother #14, first baby)

“I’m still breastfeeding this baby and if the service she offered would have been available when I had my first baby, I would have quite happily breastfeed him and it would have been quite different”. (Mother #12, second baby)

“It was lovely to be able to ask questions and gain assurance over the phone. Meeting someone one to one before the birth was very helpful as it gave me the confidence I needed”. (survey mother #234)

The peer supporters felt that women valued their input, were very appreciative and health visitors had told them that women had reported how helpful the peer supporters have been:

“It is often the little extras that have made the difference for someone – a few extra tips or an extra phone call to help a woman over a hurdle and so continue to breastfeed”.

Breastfeeding support groups in the areas included those that were well established and new ones started with women supported by the peer supporters. These groups continued the support that women needed after one-to-one peer support had stopped at two weeks.

The encouragement to attend local breastfeeding groups was also welcomed and this helped to provide the on-going support that many needed.

“There is a peer supporter at the breastfeeding group and 6 weeks on things still come up and it is really helpful to have somebody to talk to…if I didn’t have the peer supporter to talk to about things I would be more likely to give up”. (Mother #5, first baby)

“I think it is very helpful and brilliant especially for those in two minds, or maybe do not find it a natural thing, my mum bottle fed me, and it has always been like that. But to have that support just a phone call away when things are tough, to be able to phone somebody or go to the group or anything like that is how I have carried on, … if that support had not been there for me in the first couple of days, I do not think I would be doing it now”. (Mother #11, second baby, first time breastfeeding)

Peer supporters encouraged mothers to go to the groups antenatally to see what happens; to see that women breastfeed there and to get to know a few people. These groups often have an experienced peer supporter or breastfeeding counsellor attending on a rota basis each week, in addition to the volunteer supporters, so adding value to the groups.

“The groups are not getting too busy and in some places the extra mothers are helping to keep the support groups going. Some mothers prefer to see their own peer supporter when they go to a group and are a bit reluctant to go if we are not going to be there”. (PS #4)

Midwives felt that peer support should ideally last for longer than two weeks to provide active breastfeeding support over the time when many women stop breastfeeding and so attendance at support groups was important.

“Encouraging women to attend breastfeeding support groups had been a ‘real positive’ of the scheme and where peer supporters are able to attend these groups it helps to keep women breastfeeding for longer”. (MW #2)

### Challenges of peer support – partners, building trust, role conflict

The peer supporters tried to arrange for partners to attend the antenatal meeting with the pregnant women. They sold this meeting to the women as an opportunity to meet them before the baby arrived and to talk about feeding choices. Some peer supporters found it quite difficult to get partners to attend but those who did attend seemed to be interested.

“I’m struggling to get many partners to attend the antenatal visit – they are often at work or just out, but if they do attend they seem to enjoy it”. (PS #6)

When the service started, peer supporters felt that the midwives did not seem to be sure about their role. With time, relationships have improved with health professionals and they were gradually ‘trusted’ with a wider range of mothers. Peer supporters reported that the PS service was becoming accepted with invitations to go to parent craft classes and greater access to lists of pregnant mothers. Health visitors had also referred mothers to the service as they often held parent craft classes antenatally.

Midwives commented that:

“Setting up and running the service took a while as relationships, communication and trust were established, but we would be very disappointed if the service was not continued as this is a valued role within the midwifery team in our area”. (MW#2)

“The peer supporters provide a large amount of breastfeeding support and we really value the role”. (MW#3)

A few peer supporters were also trained breastfeeding counsellors (by LLL or ABM) and the distinction between a true peer support role and knowledge as a breastfeeding counsellor was blurred in some situations and this was quite challenging for some midwives, but on-going meetings were helping to sort out any conflicts in role and differences in advice.

“When peer supporters give different breastfeeding advice from our team, this could cause problems, so we encourage regular communication and always to refer clinical issues back to us”. (MW #1)

Overall midwives felt that the service was good and that this type of support was definitely needed, especially as midwives currently have so little time to spend with women.

### Breastfeeding rates

Breastfeeding initiation and 8 week rates were documented by Barnardo’s for 2011 (for those enrolled with them) and also the number of contacts made by the peer supporters over the first year. Of the 6000 births in Bristol each year around 2000 were in the 12 target wards and 1455 were referred to the peer support service in 2011; 47% (771) of these women accepted the service and were seen antenatally by a peer supporter, with the remainder declining the service once contacted or no contact had been possible. Those who had an antenatal visit received an average of 6 peer supporter contacts in the first 2 postnatal weeks; 70% were exclusively breastfeeding at 48 hours and a further 19% were partially breastfeeding (89% breastfeeding). At 2 weeks, when the one-to-one peer support finished, 54% of women who received an antenatal visit were exclusively breastfeeding and a further 17% were partially breastfeeding (71% breastfeeding).

**Figure 1:**
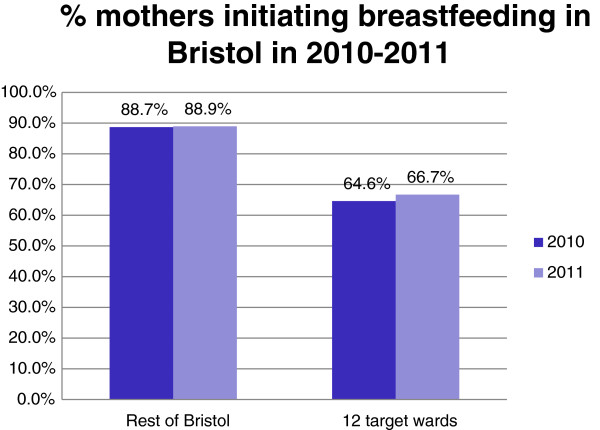
Percentage of mothers initiating breastfeeding in Bristol in 2010-2011.

**Figure 2:**
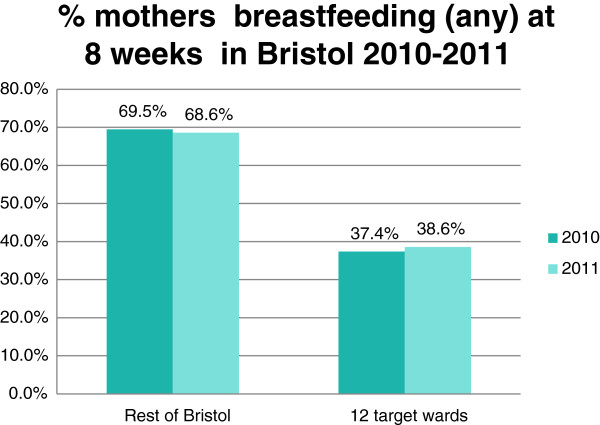
Percentage of mothers recorded as breastfeeding at 8 weeks in Bristol 2010-2011.

**Figure 3:**
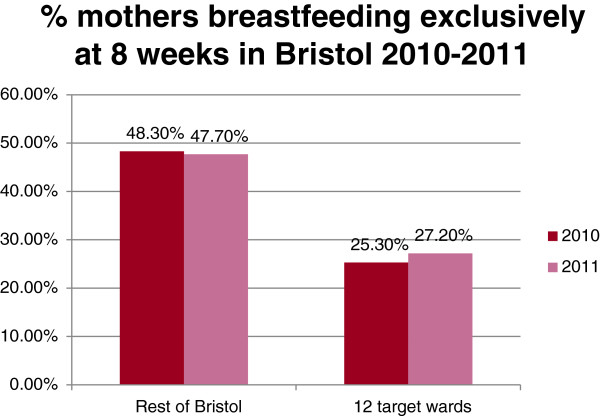
Percentage of mothers recorded as breastfeeding exclusively at 8 weeks in Bristol in 2010-2011.

The NHS Bristol routinely collected breastfeeding rates for all women in the 12 wards were compared to the rates for women in the rest of Bristol for the 12 months before and after the service started (2010 compared to 2011). There were were slight increases in breastfeeding initiation rates (2.1%) and in both total (1.2%) and exclusive breastfeeding rates(1.9%) at 8 weeks in the 12 peer support targeted wards in Bristol compared to no increases in breastfeeding rates in the rest of Bristol, as shown in the graphs in Figures 1, 2, 3. These small increases in rates in the 12 wards for 2011 were not statistically significant, but are very encouraging and show that in areas where breastfeeding is less prevalent it is possible to make small improvements in breastfeeding rates against a background of no city-wide increases. It is hoped that these small changes will continue to take place each year as the peer support service becomes embedded in the communities.

## Discussion

This study has shown that the introduction of a targeted peer support service was associated with psycho-social benefits as outlined in the positive evaluation of the service by mothers, health professionals and peer supporters. Mothers were overwhelmingly positive; felt that the peer supporters increased their confidence to breastfeed and that the service should be available to all women. Peer supporters found the antenatal visits and postnatal contacts rewarding, enjoyable, encouraging, worthwhile and important for mothers. Midwives and maternity support workers were convinced of the benefits of the peer support service, particularly the continuity of an antenatal visit and postnatal support from the same local supporter. There were also small, but non-significant increases in breastfeeding rates, and particularly exclusive breastfeeding rates, compared to the rest of the city.

The strength of the study is that it was a large mixed methods evaluation reporting the views of women, peer supporters and health professionals to describe all aspects of the process. It also included the views of women who had stopped breastfeeding within the first two weeks postnatally and who were not necessarily the ‘committed breastfeeding women’ that are often the subjects of qualitative studies. However some of those who took part may have been more likely to have been committed to breastfeeding since they were motivated to complete the on-line survey. To explore this more we interviewed women who had continued to breastfeed for several months as well as some who gave up in the early weeks.

Another limitation of the study includes the fairly low (around 40%) response rate by women to the request to complete the on-line survey, thus making the results less generalizable across the target areas. However these response rates are very similar to those found by others conducting on-line surveys [27].

In a study of one-to-one peer coaching compared to group-based breastfeeding peer support in rural Scotland very few women chose to be supported by untrained peer coaches [14]. Groups were found to be more popular because they normalised breastfeeding in a social environment with refreshments. The authors concluded that breastfeeding mothers will voluntarily engage in an activity to support breastfeeding if there is a net interactional gain and minimum risk of a negative experience. One-to one coaching was seen as a greater risk to confidence than group-based peer coaching [14]. Our study used trained paid peer supporters to provide the antenatal and immediate postnatal support and then encouraged women to access on-going breastfeeding support from the local breastfeeding groups, thus combining the best of both methods of support.

In an evaluation of a peer support service in the North West of England (Star Buddies), the strategies used by peer supporters to help women overcome obstacles were explored [9]. Through praise, reassurance and instilling calm, the peer supporters helped women achieve their goals. These phrases were echoed by the women in our study in their free text survey comments and in the interviews where they emphasised the reassurance given and how this increased their confidence to continue breastfeeding.

Our findings are also confirmed in the descriptions of the qualities needed for effective breastfeeding support reported by a metasynthesis of qualitative studies of women’s experiences of breastfeeding support [28]. An effective supporter is described by women as having an ‘authentic presence’ (showing empathy, taking time and being responsive) and a ‘facilitative approach’ (giving practical help, encouragement, realistic and accurate information); and the support service should facilitate continuity of caregiver from pregnancy to postnatal. Peer supporters in our study reported how they had provided women with “those little extras that had helped them over a hurdle and enabled them to continue to breastfeed”, which is described by Hoddinott as help being needed at ‘pivotal points’ in a woman’s breastfeeding journey and which often come at feeding transitions [29]. The skills required by peer supporters to sell the peer support service to women and provide the level of breastfeeding help needed have also been explored and described in other studies [30,31].

A lack of self-confidence in breastfeeding as a barrier to continuation has been highlighted by others [32]. Entwistle et al. [22] suggest that midwives and other health professionals supporting breastfeeding should take self-efficacy theory and the psychosocial aspects of breastfeeding support into account in their practice to help women overcome barriers of embarrassment and low self-confidence. Their study described how vicarious experience and role modelling could enhance self-efficacy and breastfeeding expectations particularly for low-income women, and this experience could be gained by providing access to peer-supporters. Comprehensive peer support initiatives that provide both one-to-one support and encouragement to attend breastfeeding support groups will help to increase breastfeeding self-efficacy and enable women to breastfeed for longer.

A systematic review of peer support interventions suggested that peer support may not be effective where routine services to support breastfeeding are well established such as in the UK [3]. Certainly antenatal peer support interventions alone do not seem to have an effect on breastfeeding rates [33], and interventions providing lay peer support only, particularly in areas where formula feeding is the norm and breastfeeding is rarely seen in the community, are not effective interventions [7]. However, continuous breastfeeding support from pregnancy and into the postnatal period using a combination of health professional and peer support may be effective in increasing breastfeeding [1,8,27].

Recent reductions in midwifery and health visiting services in the UK have resulted in much less time being available for routine antenatal education about breastfeeding and postnatal breastfeeding support. Midwives in this study valued the support for women that peer supporters were able to provide and saw them as complementary assets to the midwifery service. In areas where women have little family experience of breastfeeding and few breastfeeding role models, local peer supporters may become more important as they fulfil this role for women.

Further research could evaluate the provision of peer support schemes in different contexts and settings and track breastfeeding rates for longer as changes in infant feeding patterns take many years to achieve. In order to inform public health policies, well conducted cluster randomised control trials with peer support as part of a complex or multicomponent intervention should be encouraged, but these would be methodologically challenging and expensive to conduct.

## Conclusions

Provision of a targeted service of trained peer supporters providing antenatal and postnatal breastfeeding support was associated with small but non-significant increases in breastfeeding rates, and particularly exclusive breastfeeding rates, compared to the rest of the city. Peer support helped women feel more confident to breastfeed; peer supporters felt that the service was important and worthwhile for mothers; midwives and maternity support workers confirmed the benefits of the service, particularly the continuity of an antenatal visit and postnatal support from the same local supporter. This service continues to be offered in Bristol, but funding has to be secured on an annual basis and competes with other public health priorities. However, in areas where women have little family experience of breastfeeding, local peer supporters as described in this evaluation, working alongside health professionals, are likely to become more important in supporting women before and after childbirth.

## Competing interests

The author declares that she has no competing interests.

## Pre-publication history

The pre-publication history for this paper can be accessed here:

http://www.biomedcentral.com/1471-2393/13/192/prepub
